# COMFORTneo scale in preterm infants during live performed music therapy—Difference between close physical contact and hand touch contact

**DOI:** 10.3389/fnins.2024.1359769

**Published:** 2024-03-27

**Authors:** Susann Kobus, Tim Kleinbeck, Miriam Ader, Monia Vanessa Dewan, Anne-Kathrin Dathe, Nadia Feddahi, Ursula Felderhoff-Mueser, Nora Bruns

**Affiliations:** ^1^Department of Paediatrics I, University Hospital, University of Duisburg-Essen, Essen, Germany; ^2^Centre for Translational Neuro- and Behavioural Sciences, C-TNBS, Faculty of Medicine, University Duisburg-Essen, Essen, Germany; ^3^Center of Artistic Therapy, University Medicine Essen, Essen, Germany; ^4^Department of Health and Nursing, Occupational Therapy, Ernst-Abbe-University of Applied Sciences, Jena, Germany

**Keywords:** music therapy, Neonatology, preterm infants, behavioral state, close physical contact, hand touch contact, neonatal intensive care unit, NICU

## Abstract

There is evidence that music therapy combined with physical contact to parents stabilizes the vital signs of hospitalized preterm infants. Yet, there is no evidence for the difference between simple contact by touching the infant in the incubator or cod, or close physical contact during music therapy sessions (MT). Behavioral effects of the various forms of attention toward the infant during therapy need to be elucidated. Our study aimed to quantify the effects of hand touch contact (HTC) and close physical contact (CPC) during live performed MT in preterm infants regardless of gestational age on behavioral state (assessed via COMFORTneo scale) and vital signs. A maximum of ten live music therapy sessions were delivered three to four times a week until hospital discharge to 50 stable infants. Pre-, during- and post-therapy heart rates, respiratory rates, oxygen saturations and COMFORTneo scores were recorded for each session. A total of 486 sessions was performed with 243 sessions using HTC and CPC each. The mean gestational age was 33 + 3 weeks, with 27 (54%) infants being male. We observed lower COMFORTneo scores, heart and respiratory rates and higher oxygen saturation during and after live performed music therapy independent of the kind of physical contact than before therapy. While pre-therapy values were better in the CPC group for all four variables, a higher mean response on COMFORTneo scale and vital signs was observed for HTC (COMFORTneo score −5.5, heart rate −12.4 beats per min., respiratory rate −8.9 breaths per min, oxygen saturation + 1.5%) compared to CPC (COMFORTneo score −4.6, heart rate −9.6 beats per min., respiratory rate −7.0 breaths per min, oxygen saturation + 1.1%). Nonetheless, post-therapy values were better for all four measures in the CPC group. Regression modeling with correction for individual responses within each patient also yielded attenuated effects of MT in the CPC group compared to HTC, likely caused by the improved pre-therapy values. Live performed music therapy benefits preterm infants’ vital signs and behavioral state. During CPC with a parent, the absolute therapeutic effect is attenuated but resulting post-therapy values are nonetheless better for both the COMFORTneo scale and vital signs.

## 1 Introduction

About one in ten babies are born prematurely each year, adding up to 1.34 million worldwide (World Health Organization [WHO], 2023). Preterm infants are usually treated in the neonatal intensive care unit (NICU) for medical support immediately after birth. Painful procedures, noise of medical devices and separation from the parents create stress (Cruz et al., 2016; Yakobson et al., 2021). Recorded music reduces acute pain perception (Anbalagan et al., 2024) and shows a short-term change in physiological parameters with a decreased heart rate and increased oxygen saturation during minor painful procedures in healthy newborns (Rossi et al., 2018). In recent years, live performed music therapy (MT) as a non-pharmacological intervention to relieve stress in preterm infants in the NICU has continued to gain attention (Bieleninik et al., 2016; Haslbeck et al., 2020; van Dokkum et al., 2020; Yue et al., 2021). In preterm infants, MT improves heart rate, respiratory rate and oxygen saturation, reduces stress (Yakobson et al., 2020) and positively impacts oral feeding volumes (Loewy et al., 2013; Yue et al., 2021), potentially improving wellbeing and quality of life during the NICU stay.

During hospitalization in a NICU, parents and infants experience physical separation that can last several weeks, challenging the parent-infant closeness. Physical contact to parents provides various positive stress-relieving short- and long-term effects for preterm infants including physiological, behavioral stabilization, pain relief and parent-infant bonding (Nyström and Axelsson, 2002; Cong et al., 2009; Bahman Bijari et al., 2012; Bera et al., 2014; Bigelow and Williams, 2020; Moberg et al., 2020; Özdel and Sarı, 2020; Kobus et al., 2022a). Close physical contact after birth between parents and the infant can promote attachment between parents and infants involving both parties of the parent-infant dyade (Pennestri et al., 2015). The parental exposure to infants’ stimuli and somatosensory stimulation can improve parenting behavior and care (Strathearn, 2007). Accordingly, physical contact, with the infant lying on a parent’s arms, chest, legs or shoulders, is an important form of physical closeness encouraged in the NICU today and is associated with benefits for infants, parents, and their relationship. It accelerates the development of the infant’s sleep architecture and brain maturation (Ludington-Hoe et al., 2006; Kaffashi et al., 2013). Combining live performed MT with physical contact to parents the infant positively impacts the infants’ vital signs, promotes autonomic and physiologic stabilization reduces the length of hospital stay, and re-hospitalization rate compared to kangaroo care alone (Feldman and Eidelman, 2003; Ettenberger et al., 2016; Span et al., 2021; Kobus et al., 2022a). Exerting positive effects beyond the infant, live performed MT reduces parental and especially maternal distress and anxiety, enhancing parents’ ability to handle the stressful effects during their infant’s NICU stay (Bieleninik et al., 2016; Yue et al., 2021; Kobus et al., 2022b).

However, the interaction of different types of physical contact with live performed MT has not been investigated. The aim of this study was to compare the effects of hand touch contact (HTC) and close physical contact (CPC) between clinically stable preterm infants and a parent during live-performed MT on behavioral state, measured by COMFORTneo scale.

## 2 Materials and methods

### 2.1 Study design

The study was designed as a prospective, randomized controlled clinical trial to investigate the effects of live music therapy performed with preterm born infants and their parents. One hundred infants were randomized 1:1 to either standard medical care (control group) or standard medical care plus music therapy (intervention group). The intervention group received up to 10 sessions of music therapy, five each with CPC and five with HTC. Due to the research question of this study, only the data from the intervention group were analyzed.

### 2.2 Eligibility and recruitment

Preterm infants [gestational age (GA) <37 + 0 weeks] who were born between July 2021 and January 2023 at the University Hospital Essen were eligible to participate in the study. Exclusion criteria were clinical instability, which precluded the infant from leaving the incubator or cod, or participation in a simultaneous interventional trial. Written informed consent was obtained from all parents prior to inclusion into the study and subsequent randomization.

The study was approved by the local ethics committee of the Medical Faculty of the University of Duisburg-Essen (21-9823-BO) and registered with the German registry for clinical studies (registration number: DRKS00025755).

### 2.3 Intervention

A maximum of ten MT sessions were performed in clinically stable preterm infants in the presence of either parent three to four times per week. Before the first music therapy intervention, parents determined who, mother or father, would be present at all therapy sessions.

The timepoint of each therapy session was coordinated between music therapist, parent, and nursing staff to seamlessly integrate the session into clinical routine.

During five music therapy sessions, infants remained in the incubator or (heated) cod and the parent participating in the study touched the infant with the hand (HTC). During the other five interventions, infant and parent were in CPC, with the infant lying in the parent’s arms, on the chest, legs, or shoulder.

Hand touch contact and CPC sessions were carried out either according to clinical needs, or the type of contact infant and parent had before the start of MT. On some occasions, the type of contact was planned ahead to assure a balanced distribution between HTC and CPC sessions.

The live performed MT sessions consisted of individual, improvised playing of the instrument sansula. The instrument consists of an African kalimba attached to a drum skin, which is stretched over a wooden ring. When plucking the wooden strips on the kalimba, a vibrating, long-lasting, and soft sound is created. The therapist sat or stood next to the infant. In the first minutes of the sessions, two to three different single tones guided by the infant’s breathing and reactions were applied followed by tone sequences of a maximum of five different tones and two to three different single tones again at the end of the session. In infants with high breathing rhythm the tones were synchronized with every second or third breath to calm down the infant. After about three tones, a pause according to the length of one or two tones was used. The tempo was reduced, and the breaks were lengthened during the last 5 min of each session. The pitch lowered at the end and the final note was the deepest tone of the instrument sansula.

Music therapy was carried out individually for each infant on the NICU unit at a low volume. Behavioral state (COMFORTneo scale) and vital signs (heart rate, respiratory rate, and oxygen saturation) were recorded 10 min before, during and 10 min after each therapy session, as well as the type of physical contact during the intervention. Clinical data were retrieved from the medical records of the infants.

### 2.4 COMFORTneo scale

The COMFORTneo scale consists of seven behavioral items (alertness, calmness/agitation, respiratory response, crying, body movement, facial tension, and muscle tone). Respiratory response is applicable only to invasively ventilated infants. Each item has a score range of one to five and the total score ranges from six to thirty. A score of 14 and higher is considered as presence of distress and pain (Meesters et al., 2023). Participating infants were scored before, during (after 10 min of the intervention) and after each MT session.

### 2.5 Statistical analyses

For descriptive statistics, continuous variables are presented as median or mean and as appropriate according to the distribution of data. Discrete variables are summarized as counts and relative frequencies. Means and 95% confidence intervals (CI) were calculated for pre-, within-, and post-therapy values of the COMFORTneo scale, heart rate, respiratory rate, and oxygen saturation as well as the mean difference of pre- and post-therapy values and the percent change from baseline. The effect of the type of physical contact on the mean difference (pre- and post-therapy values) was estimated using a generalized linear mixed regression model. Because repeated measurements in the same patient are not independent, we accounted for subject-specific responses as random effect. A subset analysis was conducted to compare COMFORTneo changes depending on the parent that was present during the MT session.

SAS Enterprise Guide 8.3 (SAS Institute, Cary, CA, USA) was used to carry out analyses and produce figures.

## 3 Results

### 3.1 Patients

**FIGURE 1:**
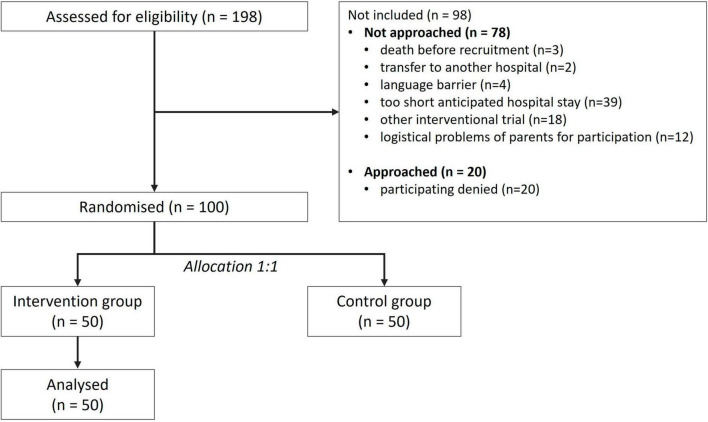
Flow chart of the included and not included patients of the study.

One hundred ninety-eight preterm infants were treated at the University Hospital Essen during the study period. One hundred infants were included into the study, 50 in the intervention group and 50 in the control group which was not analyzed in this study. Ninety-eight infants were excluded (Figure 1).

Reasons for exclusion (Figure 1) were death of the infant before recruitment (*n* = 3 [3%]), transfer to another hospital (*n* = 2 [2%]), insufficient German language skills to understand the study objectives (*n* = 4 [4%]), too short hospital stay (*n* = 39 [40%]), participating in a physiotherapy study (*n* = 18 [18%]), logistic problems of the parents for participating (*n* = 12 [12%]), and a lack of interest to participate in a study (*n* = 20 [21%]).

Excluded infants were born at a mean gestational age of 32 + 6 weeks (range 32 + 1 to 33 + 3 weeks) with a mean weight of 1970 g (range 1838 to 2101 g). Included infants were younger (32 + 1, range 31 + 4 to 32 + 6) and had lower birth weights (1751 g, range 1632 to 1870 g) (Table 1).

**TABLE 1:** Clinical characteristics of the included patients.

	Intervention group (*n* = 50)	Control group (*n* = 50)
Male, *n* (%)	27 (54)	28 (56)
GA, weeks	33.4 (± 0.5)	31.2 (± 1.0)
GA, weeks, range	26 + 0–36 + 0	23 + 2–36 + 5
Birth weight, g	1904 (± 136)	1598 (± 190)
Birth weight, g, range	425–2985	455–3630
APGAR score at 1 min.	6.8 (± 0.6)	6.7 (± 0.6)
APGAR score at 1 min, range	0.0–10.0	1.0–10.0
APGAR score at 5 min.	8.1 (± 0.5)	7.7 (± 0.5)
APGAR score at 5 min, range	1.0–10.0	1.0–10.0
APGAR score at 10 min.	8.9 (± 0.4)	8.5 (± 0.4)
APGAR score at 10 min, range	4.0–10.0	1.0–10.0
Early onset sepsis, *n* (%)	10 (20)	5 (10)
Late onset sepsis, *n* (%)	7 (14)	3 (6)
No intraventricular hemorrhage, *n* (%)	13 (26)	10 (21)
Intraventricular hemorrhage I-II, *n* (%)	37 (66)	27 (55)
Intraventricular hemorrhage III-IV, *n* (%)	4 (8)	12 (24)
Patent ductus arteriosus (total), *n* (%)	14 (28)	27 (55)
Surgery, *n* (%)	3 (6)	4 (8)
Antibiotic treatment, days	3 (± 1.5)	2.6 (± 1.5)

GA, gestational age. Data are presented as mean and standard deviation, if not indicated otherwise.

### 3.2 Music therapy sessions

A total of 486 music therapy sessions were performed with a mean duration of 28.49 min (range 21–33 min). A total of 243 HTC sessions and 243 CPC sessions were conducted. HTC sessions had a mean duration of 28.5 min (range 21–33 min) and CPC sessions 28.4 min (range 22–33 min). In forty-three children (86%) the mothers and in seven children (14%) the fathers were present. The other parent was not present during the music therapy sessions. Forty-five of 50 (90%) patients in the intervention group received 10 sessions of music therapy. Five infants were discharged unexpectedly early before the completion of ten sessions. Of these, four infants received eight sessions and one infant received four sessions. All infants received the same amount of HTC and CPC sessions. In 406 sessions the mothers were present and in 80 sessions the fathers were present.

### 3.3 Behavioral response

**FIGURE 2:**
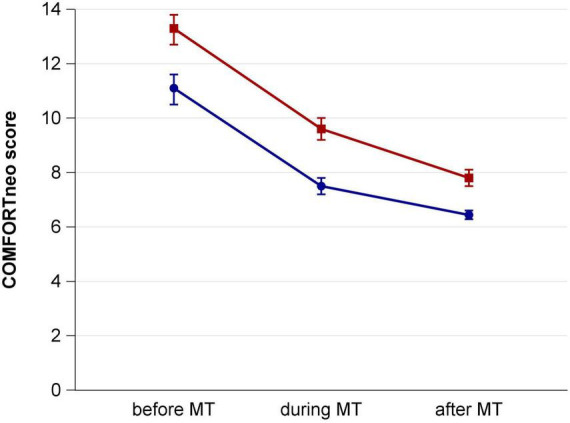
COMFORTneo score before, during and after music therapy sessions with hand touch contact (red) and close physical contact (blue).

COMFORTneo scores decreased, regardless of the type of contact the infants had to their parents during MT sessions. During therapy vital parameters were lower than before therapy and following therapy they were even lower. COMFORTneo scores were lower at all assessed timepoints if music therapy was performed with CPC compared to HTC (Figure 2).

In HTC sessions, the mean decline of COMFORTneo score was larger compared to CPC but remained at a higher post-therapy level, nonetheless (Table 2). The change from baseline was 41% in both groups. The effect of CPC on the mean COMFORTneo score pre- and post-therapy difference by regression modeling was 0.9 (95% CI 1.6–0.2) points weaker compared to HTC.

**TABLE 2:** COMFORTneo score before, during and after music therapy sessions with hand touch contact (HTC) and close physical contact (CPC) to parents.

	Contact	Sessions (*n*)	Mean before therapy (95% CI)	Mean during therapy (95% CI)	Mean after therapy (95% CI)	Mean difference (before–after) (95% CI)	% change from baseline
Overall	Close physical contact	243	11.1 (10.5–11.6)	7.5 (7.2–7.8)	6.4 (6.3–6.6)	−4.6 [−5.2−(−4.1)]	−41%
	Hand touch contact	243	13.3 (12.7–13.8)	9.6 (9.2–10.0)	7.8 (7.5–8.1)	−5.5 [−6.0−(–5.0)]	−41%
Mothers	Close physical contact	203	10.9 (10.3–11.5)	7.4 (7.1–7.7)	6.4 (6.2–6.6)	−4.5 [−5.1−(−4.0)]	−41%
	Hand touch contact	203	13.1 (12.5–13.7)	9.6 (9.2–10.0)	7.8 (7.5–8.1)	−5.3 [−5.9−(−4.8)]	−40%
Fathers	Close physical contact	40	11.7 (10.2–13.1)	8.1 (7.3–9.0)	6.7 (6.2–6.6)	−5.0 [−6.5−(−3.6)]	−43%
	Hand touch contact	40	14.1 (12.7–15.5)	9.7 (8.6–10.8)	7.7 (6.8–8.5)	−6.5 [−7.7−(−5.2)]	−46%

CI, confidence interval.

When differentiating between the presence of mothers and fathers, the mean values before therapy were higher when fathers were present than when mothers were present. With the presence of the fathers, there was a larger difference between the values before and after the session (Table 2).

### 3.4 Vital sign response

Similar results were achieved regarding vital signs (heart rate, respiratory rate and oxygen saturation). Regardless of the type of contact to the parents, the infants’ values improved during and additionally after the music therapy session. Baseline heart and respiratory rates were lower and baseline oxygen saturation was higher before CPC sessions than HTC sessions (Table 3). Like for the COMFORTneo score, the mean response to music therapy was slightly stronger in infants with HTC compared to infants with CPC, but music therapy with CPC resulted in better post-therapy values than music therapy with HTC (Table 3). The % changes from baseline were slightly stronger in the HTC group for all vital signs. Regression modeling showed that the effect of CPC on the mean pre- and post-therapy difference compared to HTC was 2.7 (95% CI 4.8–0.7) beats per minute weaker for the heart rate, 1.8 (3.2–0.5) breaths per minute weaker for the respiratory rate, and 0.4 (0.1–0.8) percentage points weaker for oxygen saturation.

**TABLE 3:** Heart rate, respiratory rate and oxygen saturation before, during and after music therapy sessions with hand touch contact (HTC) and close physical contact (CPC) to the parents.

Vital sign	Contact	Sessions (*n*)	Mean before therapy (95% CI)	Mean during therapy (95% CI)	Mean after therapy (95% CI)	Mean difference (before–after) (95% CI)	% change from baseline
Heart rate (beats/min)	Close physical contact	243	150.5 (148.6–152.5)	141.8 (139.8–143.8)	140.9 (138.7–143.1)	−9.6 [−11.1−(−8.2)]	−6.4%
	Hand touch contact	243	158.9 (157.2–160.5)	149.5 (148.1–150.8)	146.5 (145.1–147.9)	−12.4 [−14.0−(−10.7)]	−7.8%
Respiratory rate (breaths/min)	Close physical contact	243	44.0 (42.9–45.0)	38.3 (37.0–39.5)	36.9 (35.6–38.3)	−7.0 [−8.2−(−5.9)]	−15.9%
	Hand touch contact	243	51.4 (50.0–52.8)	45.1 (44.0–46.1)	42.5 (41.5–43.5)	−8.9 [−10.1−(−7.6)]	−17.3%
SaO_2_ (%)	Close physical contact	243	97.0 (96.7–97.4)	98.0 (97.8–98.3)	98.1 (97.8–98.4)	1.1 (0.8–1.3)	1.1%
	Hand touch contact	243	95.8 (95.4–96.2)	97.0 (96.7–97.4)	97.3 (97.0–97.6)	1.5 (1.2–1.8)	1.6%

CI, confidence interval; SaO_2_, oxygen saturation.

## 4 Discussion

The present study revealed that music therapy improves preterm infants’ behavioral state measured via the COMFORTneo scale. Lower COMFORTneo scores after MT were observed independently of the type of physical contact between infants and their parents during the intervention. Pre-therapy COMFORTneo scores were lower in infants who had CPC with their parents prior to MT, resulting in lower absolute values at all-time points but—possibly due to improved baseline values—a weaker therapeutic effect of MT. The same findings were observed for heart rate, respiratory rate, and oxygen saturation. A subgroup analysis showed slightly stronger decreases of COMFORTneo scores if fathers were present during the session compared to mothers.

The findings on improved vital signs confirm previous study results that live performed music therapy has positive effects on heart rate, respiratory rate and oxygen saturation (Loewy et al., 2013; Kobus et al., 2021, 2022a). Only few randomized controlled trials with preterm infants have investigated physical contact to their parents with or without MT (Teckenberg-Jansson et al., 2011; Yakobson et al., 2020). These studies focused on vital signs and maternal anxiety rather than the behavioral state of the infant (Teckenberg-Jansson et al., 2011; Bieleninik et al., 2016). A decreased heart rate and respiration and increased transcutaneous O_2_ saturation were observed when music therapy was delivered in combination with CPC (Teckenberg-Jansson et al., 2011; Kobus et al., 2022a).

It is generally acknowledged that close physical contact improves the behavioral state of preterm and term born neonates, including routine care such as feeding and during invasive procedures (Bera et al., 2014; Özdel and Sarı, 2020; Todil and Cetinkaya, 2022) and is beneficial for the preterm infants and their parents (Bedetti et al., 2023). Different kinds of physical contact exert different effects on preterm infants: dynamic touch reduced physiological arousal and decreases heart rates until 5 min after the touch in randomized controlled study. In contrast, static touch did not lead to a significant change in the heart rates of preterm infants. Dynamic touch was associated with an increase in oxygen saturation, which was not observed in the infants who received static touch (Manzotti et al., 2019). In our study, static touch was used but enhanced with music therapy, thereby providing a different type of additional stimulus during physical contact. So far, very few studies have focused on the influence of MT on preterm infants’ behavioral states. Two studies investigating the influence of recorded music on preterm infants’ behavior found no beneficial effects (Arnon et al., 2006; Alipour et al., 2013). A study with preterm infants born before 32 weeks of gestation showed that live performed lullabies had a larger beneficial impact on their sleep state than recorded lullabies (Garunkstiene et al., 2014). In these studies, however, it was not reported whether the infants had physical contact to their parents during the intervention. The present study is the first to investigate the impact of the type of physical contact by the parents on the COMFORTneo scale in preterm infants. Our findings expand the understanding on how the combination of MT with non-pharmacological stress-reducing measures achieve additional effects regarding both vital sign and behavioral stabilization.

Further, we found slightly higher baseline COMFORTneo scores if fathers were present during the session compared to mothers. The observed effects of music therapy during these sessions were slightly stronger in fathers than in mothers. While most MT studies on infant and parental responses involve mothers, these results show that also fathers’ roles deserve to be investigated in the context of NICU music therapy (Feldman et al., 2019; Baldoni et al., 2021).

The major limitation of our study is that there was no placebo intervention, making it impossible to track the spontaneous evolution of the COMFORTneo score over a time period equivalent to the duration of a music therapy session. Future trials should provide a placebo intervention addition in order to have an additional source for comparison when assessing the effect of MT. Changes in oxygen supplementation were not recorded during therapy sessions. In addition, sessions were performed at different times of the day and the type of physical contact followed no strict protocol, potentially leading to varying intra- and interindividual response profiles caused by environmental circumstances.

Our study provides new evidence that MT is an effective measure to reduce preterm infants’ distress regardless of the type of physical contact with a parent. Closer contact was associated with even lower overall COMFORTneo scores at all-time points and at both parents, mothers, and fathers. Thus, combining non-pharmacological stress-reducing strategies can enhance the total effect of MT.

## 5 Conclusion

In summary, this study provides evidence that CPC of preterm infants with their parents during live performed MT interventions adds up to lower post-therapy COMFORTneo scores and better absolute vital sign values compared to HTC. However, even if the infant cannot be removed from the incubator or cod, MT with additional HTC clearly influences the infant’s behavioral state in a positive way.

## Data availability statement

The original contributions presented in the study are included in the article/Supplementary material, further inquiries can be directed to the corresponding author.

## Ethics statement

The study was approved by the local Ethics Committee of the Medical Faculty of the University of Duisburg-Essen (21-9823-BO) and registered with the German registry for clinical studies (registration number: DRKS00025755). Written informed consent was obtained from all parents prior to inclusion into the study and subsequent randomization.

## Author contributions

SK: Conceptualization, Data curation, Formal analysis, Funding acquisition, Investigation, Methodology, Project administration, Resources, Software, Supervision, Validation, Visualization, Writing – original draft, Writing – review and editing. TK: Writing – review and editing. MA: Writing – review and editing. MVD: Writing – review and editing. A-KD: Writing – review and editing. NF: Writing – review and editing. UF-M: Writing – review and editing. NB: Writing – review and editing.
