# Supplementary material for: COMFORTneo scale in preterm infants during live performed music therapy—Difference between close physical contact and hand touch contact
Source: Front Neurosci. 2024 Mar 27;18:1359769. doi: 10.3389/fnins.2024.1359769 (PMC11008230; doi:10.3389/fnins.2024.1359769)
Supplement: Supplementary file 1 [file Table_1.pdf]

Table S1. Clinical characteristics of excluded patients.

	**Excluded patients (n = 98)**
Male, n (%)	55 (56)
GA, weeks	32.8 (±0.6)
GA (weeks), range	23+4–36+6
Birth weight, g	1970 (±132)
Birth weight (g), range	400–3490
Apgar score at 1 min.	7.1 (±0.5)
APGAR score at 1 min, range	0.0–10.0
Apgar score at 5 min.	8.2 (±0.4)
APGAR score at 5 min, range	1.0–10.0
Apgar score at 10 min.	8.8 (±0.3)
APGAR score at 10 min, range	1.0–10.0
Early onset sepsis, n (%)	14 (14)
Late onset sepsis, n (%)	7 (7)
No intraventricular hemorrhage, n (%)	35 (36)
Intraventricular hemorrhage °I-II, n (%)	50 (51)
Intraventricular hemorrhage °III-IV, n (%)	14 (14)
Patent ductus arteriosus (total), n (%)	37 (37)
Surgery, n (%)	4 (4)
Antibiotic treatment, days	2.8 (±1.7)

Notes. GA = gestational age. Data are presented as mean and standard deviation if not indicated otherwise.
